# Streamflow in the United States: Characteristics, trends, regime shifts, and extremes

**DOI:** 10.1038/s41597-024-03618-0

**Published:** 2024-07-17

**Authors:** Yiming Wang, Xuesong Zhang, Kaiguang Zhao, Debjani Singh

**Affiliations:** 4 Oak Ridge Institute for Science and Education, TN Oak Ridge, 37830 USA; 1 USDA-ARS Hydrology and Remote Sensing Laboratory, Beltsville, MD 20705-2350 USA; 2 Ohio Agricultural Research and Development Center, School of Environment and Natural Resources, The Ohio State University, Wooster, OH 44691 USA; 3 Environmental Sciences Division, Oak Ridge National Laboratory, Oak Ridge, TN 37831 USA

**Keywords:** Hydrology, Environmental impact

## Abstract

Long-term streamflow observations contain essential information for understanding hydrological changes and managing water resources. A continental-scale dataset or analysis of temporal streamflow change is still lacking across hydrologic gauges in the Conterminous United States (CONUS). Here, we compiled 70 years of streamflow records from 1951 to 2021 at ~ 8000 hydrologic stations across the CONUS and characterized temporal trends, regime shifts, and extreme events using a Bayesian time series analysis algorithm. We found that the occurrences of sudden streamflow changes (e.g., regime shifts and extreme events) have been increasing with time across the CONUS. In addition, we derived 181 streamflow indicators that are valuable for hydrological and biological applications, such as the duration and frequency of high or low streamflow events. The Mississippi River Basin, especially the middle and lower parts, was a hot spot of high-frequency high-flow events. Overall, we anticipate the dataset generated here offers valuable information for understanding and quantifying changes in water resources across the CONUS.

## Background & Summary

Streamflow is a fundamental component of the hydrological cycle and a cornerstone of freshwater resources. Understanding changes in streamflow provides useful information for ensuring water availability and ecosystem health^1^. Spatial and temporal variations in streamflow are driven by natural and anthropogenic factors such as climate change (e.g., warming and enhanced evapotranspiration) and human interventions (e.g., irrigation and land management). Both theoretical evidence and model simulations suggested an intensification of wet and dry anomalies in streamflow since the twentieth century, attributed primarily to anthropogenic drivers^2^. In the Conterminous United States (CONUS), streamflow in the near future (2021–2050) was projected to deviate significantly—up to 30%—from the 1980–2010 averages^3,4^. Tracking or predicting such changes is contingent upon not just the predictive skills of models but also the reliable synthesis of historical *in-situ* observations.

Despite the availability of stream gauge data, there still exists a strong need for better synthesis of long-term changes in streamflow over large geographic regions. This is especially the case for streamflow characteristics with ecological and biological implications. In the U.S., multiple existing streamflow datasets either relied on localized modelled streamflow or focused only on metrics associated with specific land cover types^5–8^. In addition, streamflow characteristics often vary nonlinearly across a spectrum of time scales, making it challenging to characterize its long-term pattern. In this regard, another critical gap is the lack of a multi-scale analysis of trends, seasonality, and regime shifts in streamflow. Filling this gap is not only valuable for scientific curiosity but also imperative for strategic planning^9^, which stresses the need for new datasets to facilitate the comprehensive understanding and quantification of long-term streamflow variations over the CONUS.

Characterizing streamflow dynamics and trends often use parametric and nonparametric statistical approaches. Common methods include the Mann-Kendall test^10–12^, the Pettitt test^13,14^, and the Chow test^15–17^. Although these classical methods demonstrated certain robustness and practical utility, they have limitations of all kinds, such as restrictive model assumptions, difficulties in handling an unknown number of changepoints, failure to differentiate seasonal from secular variations, and the inability to model nonlinear trends. Many of the limitations have been remedied by recent advances in time series analysis algorithms. One new algorithm is the Bayesian estimator of abrupt change, seasonal change, and trend (BEAST)^18^. It incorporated the state-of-the-art Bayesian computation to accommodate uncertainties of all sorts in decomposing time series into separate components^18^. As a generic algorithm, its usefulness and superiority have been exemplified in applications for dozens of disciplines (e.g., ecology, climate sciences, public health, and medical sciences). BEAST proved useful for probing mechanistic drivers of trends or abrupt changes in long-term time series data. Leveraging the most current state-of-the-art time series algorithms such as BEAST is expected to glean new insights unattainable by classical methods.

**Fig. 1:**
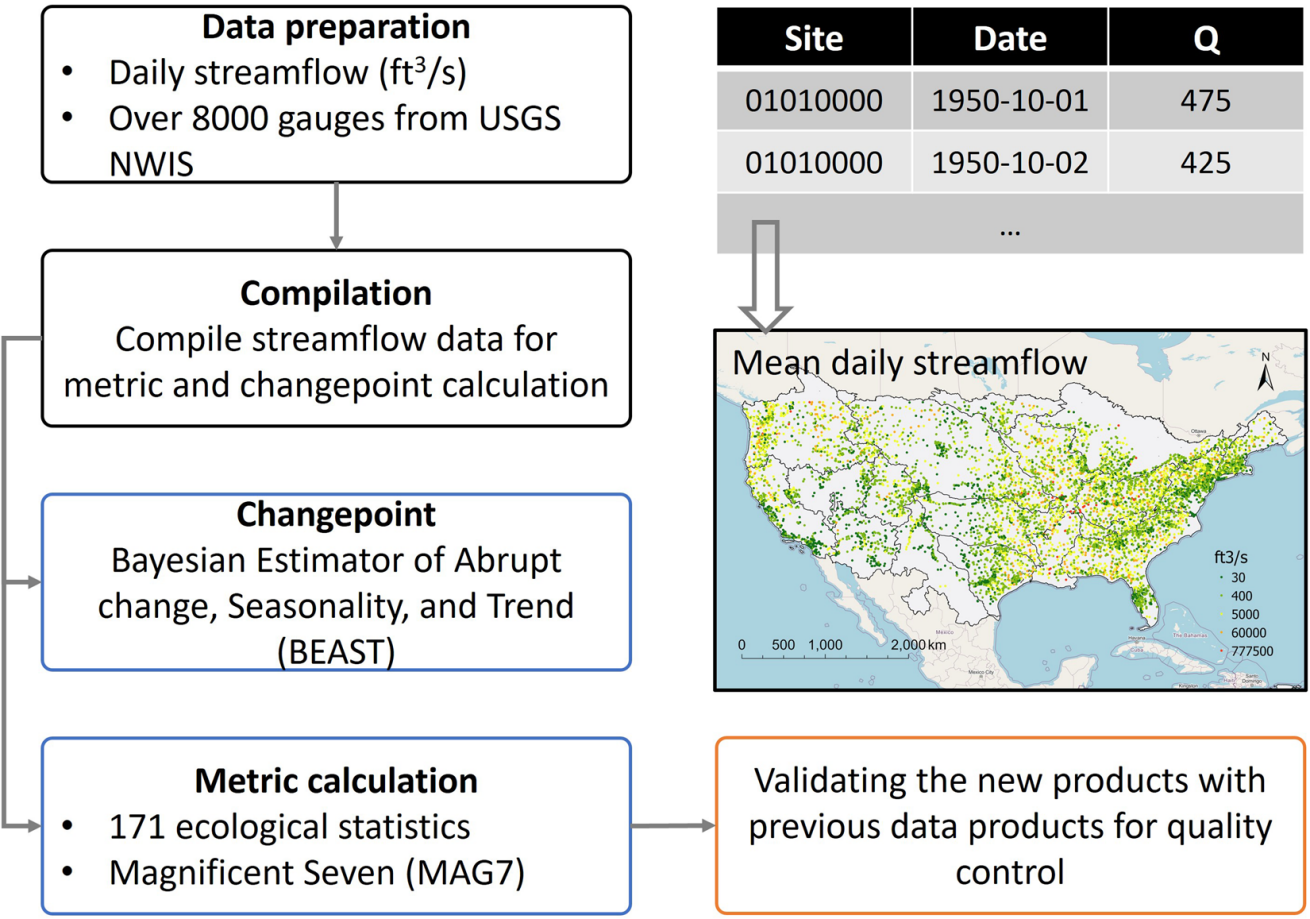
The workflow for deriving streamflow trends and characteristics across the CONUS.

Here, we generated a comprehensive dataset of 181 streamflow metrics and identified changepoints in streamflow patterns for over 8,000 hydrological stations, with drainage areas ranging from 0.026 to nearly 3,000,000 km^2^, spanning the CONUS (Fig. 1). To create this dataset, we first gathered daily streamflow observations from the U.S. Geological Survey (USGS) National Water Information System (NWIS) and processed them using the R programming language. Subsequently, we calculated 181 streamflow statistics utilizing the EflowStats package. To ensure the reliability and accuracy of these derived streamflow metrics, validation was undertaken by comparing with the Catchment Attributes and Meteorology for Large-sample Studies (CAMELS) dataset. Additionally, we employed the BEAST method to identify changepoints and outlier years of the time-series streamflow data. This new dataset, comprising the streamflow metrics and changepoints, is expected to contribute significantly to our understanding of ecological conditions within river systems and provide an independent dataset for the validation of hydrological models. Detailed descriptions of the key methods and procedures employed in developing this dataset are presented in the Methods section.

## Methods

### Data compilation

We accessed daily streamflow data from the USGS NWIS using the dataRetrieval package in R^19^. This extensive dataset encompasses records from over 15,000 monitoring stations, providing comprehensive coverage of the CONUS. We filtered the observed streamflow data based on the USGS daily value qualification codes, focusing on records spanning the period from 1951 to 2021. To ensure the representation of robust long-term streamflow characteristics and changes, we excluded stations with less than 5 years of available data. After these filtering steps, we organized all the retained streamflow data in R. This refined dataset, comprising 8,269 stations with drainage area ranging from 0.026 to 2,926,687 km^2^ encompassing small catchments to large watershed outlets, served as the foundation for generating streamflow metrics and identifying changepoints in streamflow patterns.

### Streamflow statistics

We derived streamflow statistics from daily streamflow data using the EflowStats package in R^20^, including biologically relevant streamflow statistics^21^ and the “Magnificent Seven” (MAG7)^22^ indicators. The biologically relevant statistics consist of 171 indicators, categorized into five groups: (i) magnitude, (ii) frequency, (iii) duration, (iv) timing, and (v) rate of streamflow changes. For magnitude, we considered both low and peak flow conditions. For frequency, duration, and timing, we also computed metrics for both low and high flow events under various threshold scenarios, such as using the average number of flow events with flows above different thresholds. It’s worth noting that when describing the duration of high flow events, we utilized a flood recurrence interval of 1.67 years and the median or percentile flow values as the flood threshold^23^.

The MAG7 statistics include seven fundamental properties of daily streamflow data: (i) mean; (ii) coefficient of variation; (iii) skewness; (iv) kurtosis; (v) autoregressive lag-one correlation coefficient; (vi) amplitude; and (vii) phase of the seasonal signal. As elucidated by Archfield *et al*.^22^, these metrics effectively capture the statistical characteristics of streamflow and the correlated nature of its time series.

To ensure consistency across all sites and simplify the process, we employed the calendar year rather than the water year when generating these statistics. Apart from the year type, all other parameters are set to their default values. Additionally, beyond the commonly recommended indices, we also derived the 5% and 95% quantiles of streamflow, which were used to evaluate the robustness of the metrics derived from this study as compared against previous products.

### Time series analysis for trends, changepoints, and extremes

We characterized streamflow dynamics via time series decomposition to identify trends, changepoints (e.g., regime shifts), and extreme events using the Bayesian estimator of abrupt change, seasonal change, and trend (BEAST)^18^. BEAST is a robust and versatile Bayesian model averaging technique for detecting changes, trends, and periodic variations^18,24^. It has been empirically demonstrated as a powerful tool for analysing long-term observations of hydrological processes and water quality^25^.

**Fig. 2:**
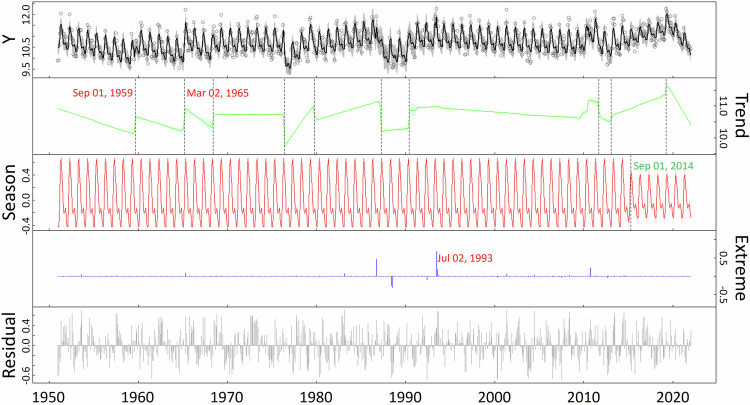
Illustration of BEAST decomposition for detecting changepoints and extreme events. An example shows regime shifts of streamflow at the gauge 05420500 in the Upper Mississippi River. Y represents the natural logarithm of daily streamflow.

The BEAST method accounts for temporal variations at multiple scales by treating a streamflow time series $y(t)$ as the composition of four components–seasonality, trend, extremes/outliers, and residuals:

1 $$y\left(t\right)=S\left(t{\rm{;}}{\Theta }_{S}\right)+T\left(t{\rm{;}}{\Theta }_{T}\right)+E\left(t{\rm{;}}{\Theta }_{E}\right)+\varepsilon $$

where the seasonality $S$ encapsulates seasonal/periodic patterns; the trend $T$ captures the secular change; the outlier component $E$ indicates extreme events occurring at individual points of time; and the residuals ${\varepsilon }_{i}$ are random Gaussian model errors. Both the $S$ and $T$ components may contain sudden changes at certain times, defined as seasonal or trend changepoints at which seasonal or trend signals start to deviate from the previous regular trajectories (e.g., vertical dashed lines in Fig. 2). As such, $S$ and $T$ are parameterized as piece-wise harmonic and piecewise linear functions, respectively. The locations of the changepoints and extreme events are denoted by the ${\Theta }_{S}$, ${\Theta }_{T}$, and ${\Theta }_{E}$ parameters in Eq. 1. Using the trend $T$ as an example, a changepoint refers to a sudden change in slope, intercept, or both. We inferred the different components and their associated changepoints using the Rbeast package in R (https://github.com/zhaokg/Rbeast). Because no prior information on the numbers and locations of the changepoints was available, we used a flat prior in the BEAST modelling, with more detailed in Table S2. More technical information about BEAST are available in Zhao *et al*.^18^.

BEAST offers a rich set of streamflow statistics unobtainable from traditional time series analysis methods (Fig. 2). As a Bayesian method, it quantifies uncertainties of all kinds. An example of typical outputs from BEAST is given in Fig. 2 for the streamflow data at the gauge 05420500 in Upper Mississippi River. BEAST decomposed the time series into distinct components: trend, seasonality, and outliers/extremes. In the trend component, BEAST identified 10 changepoints, together with the changepoint occurrence probability over time. These changepoints were mostly associated with synoptic climate drivers. For example, the changepoint in 1959 is attributed to the cessation of drought in the U.S. Midwest^26,27^, whereas the shift in 1965 is associated with the Spring Flood occurring in April and May^28,29^. Moreover, the BEAST also detected extreme event of summer flooding in 1993^30^.

## Data Records

**Fig. 3:**
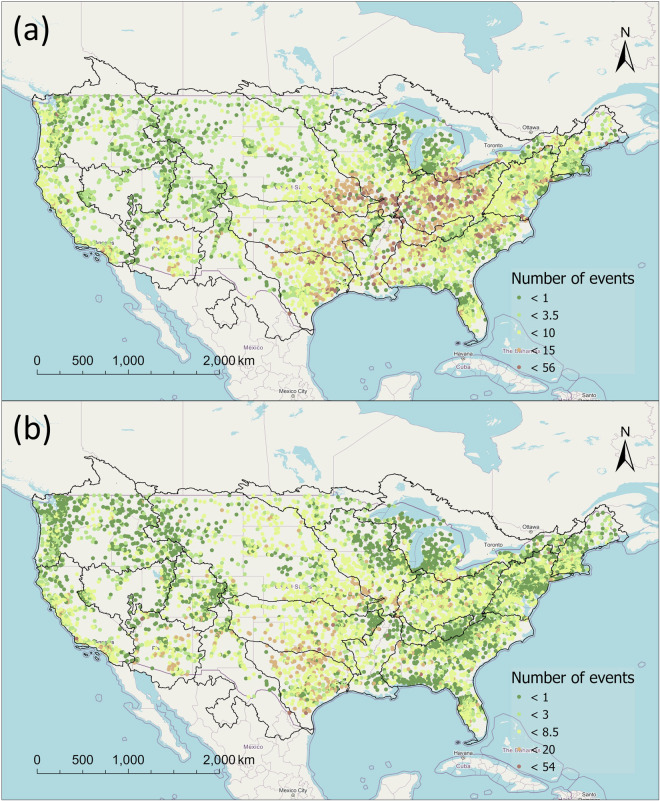
The frequency (number of events per year) of (**a**) high flow events and (**b**) low flow events in the CONUS. The high flow here was based on the flow above a threshold that is equal to seven times the median flow value for the entire flow record. The low flow is the average pulse duration (table S1) for flow events below a threshold that is equal to the 25^th^ percentile value for the entire flow record. The larger point size represents the longer duration.

The newly developed streamflow characteristics and changes dataset is available at figshare^31^, consisting of two sub-datasets: (1) “Streamflow characteristics” and (2) “Changepoints and Extremes of Streamflow”. The “Streamflow characteristics” dataset comprises a total of 181 streamflow metrics for each of the 8,354 gauges, as detailed in Table S1. Within this dataset, the highest recorded mean daily streamflow values were observed in the lower regions of the Mississippi River, with values reaching as high as approximately 21,000 m^3^ s^−1^ (Fig. 1). Furthermore, there is a discernible spatial pattern when examining the frequency and duration of high flow events (Fig. 3a). In general, the Midwest region exhibits a more frequent high flow events compared to other regions within the CONUS. Notably, a concentration of high-frequency high flow events was observed near the outlets of the Missouri Region, Upper Mississippi Region, and Ohio Region. Additionally, high flow events are also frequent in Texas-Gulf Region and Lower Mississippi Region. In contrast, the spatial pattern of low flow events appears more dispersed, with most stations experiencing fewer than 10 occurrences per year (Fig. 3b). Moreover, we have provided a separate table that offers information on the number and timespan of available observations at each station. This allows users to select hydrological stations that align with their specific criteria, catering to various analytical needs.

**Fig. 4:**
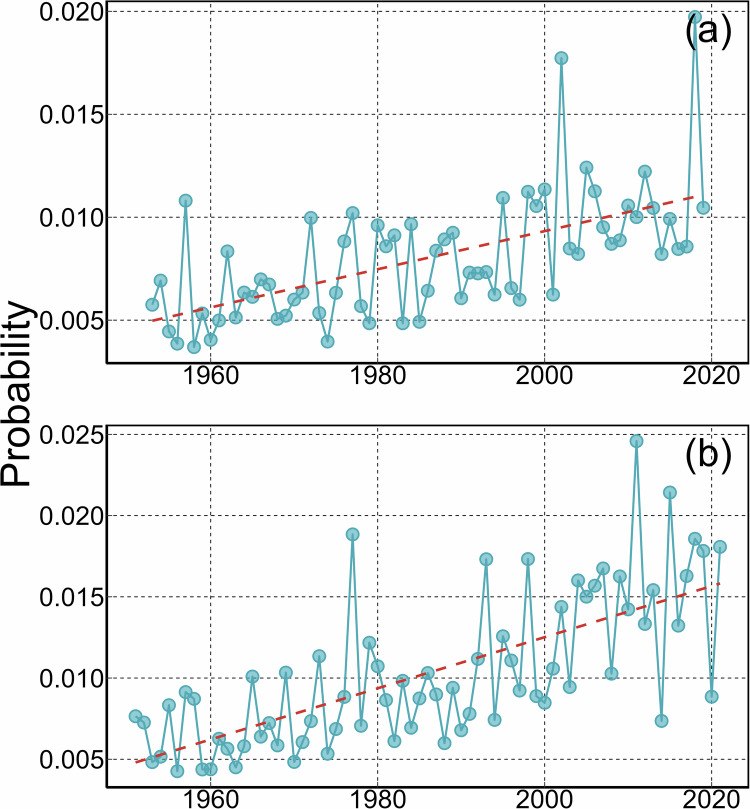
Increased probabilities over time in observing (**a**) regime shifts (i.e., changepoints in trend) and (**b**) extreme streamflow events across the CONUS. The probability curves are the average across all stations.

The “Changepoints and Extremes” dataset provides information on the most probable years of streamflow change and the associated probabilities of these changepoints. This dataset encompasses changes in long-term trends, seasonal components, and outlier events, as outlined in Table 1. Importantly, this dataset retains all 8,269 stations, with results labelled as “NA” indicating stations with no significant changes after 1951. To facilitate user-friendly data filtering, we also included additional metrics at each station, including the average marginal likelihood, R-square, root mean square error (RMSE), and the estimated variance of the model error, which show how the average fit of a model to a data set. In general, the higher marginal likelihood and R-square, and lower value of RMSE and variance of the model error indicates a better accuracy of results. One example of using this dataset is examining the years of changes. In general, there is a notable increase in the possibility of streamflow changes and extreme events after the year 1960 over the CONUS, indicating a higher fluctuation in recent years (Fig. 4). These more intense changes in streamflow could be attributed to climate change or anthropogenic influences^32–34^. Furthermore, the dataset reveals two significant and abrupt fluctuations of extreme streamflow events since 1951. The first notable shift occurred around 1977, followed by a second substantial change near 2011. But overall, the increases in the possibility of extreme events could be attributed to heightened occurrences of extreme climate events such as heavy rainfall and droughts^34–36^.

**Table 1:** Data records contained in the “Changepoints and Extremes” dataset.

Field	Definition
Station ID	U.S. Geological Survey designated ID
drainArea	Drainage area of USGS stations (km^2^)
Changepoints	The most probable decimal dates of streamflow change
cpPr	The possibility of changepoints
cpQ	The discharge value at the changepoint dates (m^3^/s)
Season	The most probable decimal dates of seasonal trend changes
seasonPr	The possibility of seasonal trend changes
Outlier	The most probable decimal dates of streamflow outliers
outlierPr	The possibility of outliers
outlierQ	The discharge value at the outlier dates (m^3^/s)
lik	The average marginal likelihood
r2	R-square
rmse	Root mean square error
sig2	The estimated variance of the model error
pos_ncp	Number of positive changepoints
neg_ncp	Number of negative changepoints

## Technical Validation

**Fig. 5:**
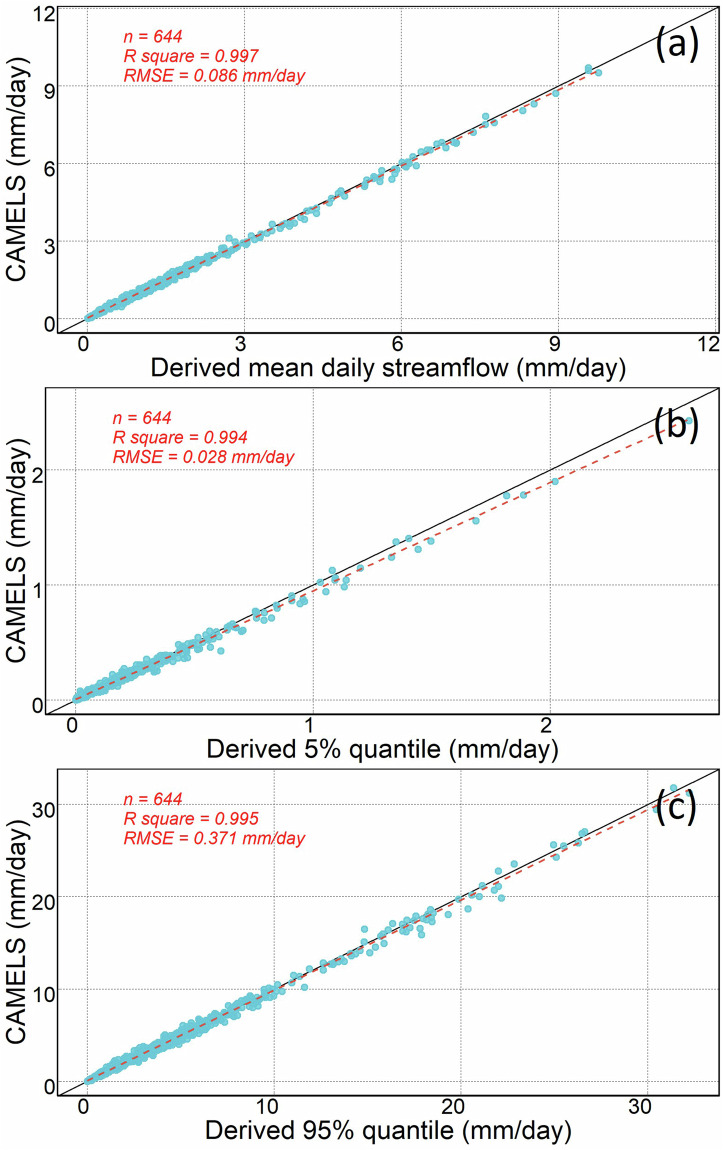
Comparison between CAMELS and streamflow statistics derived in this study for (**a**) mean daily streamflow, (**b**) 5% streamflow quantile, and (**c**) 95% streamflow quantile. The black solid line indicates 1:1 relationship, and the red dashed line is the regression line. Each blue dot represents the variable of interest at a USGS station.

We validated the derived streamflow statistics by comparing against an independent daily streamflow dataset--CAMELS^37^ and a sub-daily flooding ground-based flood dataset^38^. The CAMELS dataset covers 671 basins across the CONUS, with mean daily streamflow data spanning from October 1, 1989, to September 30, 2009. To validate the accuracy and reliability of our dataset, we conducted a comparative analysis of streamflow statistics from 644 USGS stations. The comparison encompassed mean daily streamflow, the 5% and 95% streamflow quantiles. The results show a high degree of correlation and low RMSE between the CAMELS dataset and streamflow statistics derived in this study (Fig. 5). Specifically, for these three indices, R-square is greater than 0.99, and RMSE remains below 0.4 mm/day, indicating a robust relationship and minimal variance of residuals. Most data points closely align with the 1:1 line. The slight divergence may be attributed to disparities in data periods. Our statistics, derived from a longer data period, can be influenced by ongoing climate change, which has been known to impact the magnitude of river flows^39^.

**Fig. 6:**
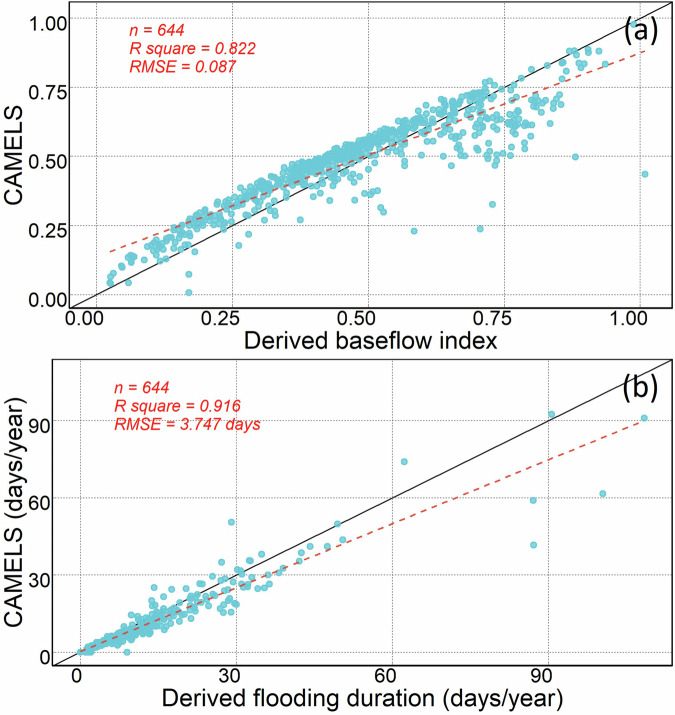
Comparison between the CAMELS and derived streamflow statistics in this study for (**a**) baseflow index and (**b**) flood duration.

In addition to the above metrics, we also conducted evaluations for the baseflow index and flood duration time, comparing hydrological signatures from the CAMELS dataset with our dataset (Fig. 6). The baseflow index, representing the ratio of mean daily baseflow to mean daily streamflow, exhibits a high level of agreement between the two datasets. The overall R-squared value is higher than 0.8, while RMSE is lower than 0.1. Most data points closely align along the 1:1 line, with minor deviations observed at a few stations. These discrepancies may be attributed to variations in the data periods utilized.

Flood duration, which measures the average duration of high-flow events, also demonstrates a strong agreement between the two datasets. It is essential to note that there are methodological differences in estimating flood duration between the two datasets. In the CAMELS dataset, the duration of high-flow events was calculated based on the number of days when daily streamflow was nine times higher than the median daily streamflow. In contrast, we determined high-flow events using streamflow values above seven times the median daily streamflow. As a result, our dataset generally yields slightly higher high-flow durations, particularly for stations with extended high-flow events. Nevertheless, the overall agreement between the two datasets remains high, with R-square exceeding 0.9.

We also found that the high flow duration derived from the daily flow records generally agree with that of the fine-resolution US flood database^38^ (Fig. S1). The deviation between these two datasets may arise from the difference in resolution of the dataset (i.e., daily vs. sub-daily), periods of available data, and sources of the data.

## Usage Notes

The methods and data records sections provide a comprehensive primer for interested readers to understand how the metrics were derived and organized. This will facilitate informed utilization of the dataset for their research endeavours. Furthermore, users have the flexibility to choose a subset of the CONUS scale dataset that aligns with their specific research goals and study areas on a smaller scale. It’s important to note that the available time periods of observed streamflow data vary at different hydrological stations. Hence, we highly recommend that users select hydrologic stations that contain data representative of the period of their study.

We envision the new dataset generated in this study can be applied in a wide range of research areas, including but not limited to the following:

1. Metrics from the “Streamflow Statistics” dataset, such as average and peak streamflow, can serve as valuable indicators for validating process-based hydrological models. These derived indicators are particularly useful for evaluating the spatial patterns generated by watershed models, offering insights into the average conditions at hydrological stations along river channels.
2. The “Streamflow Statistics” dataset can also be integrated with other datasets, such as climate, land use, and soil properties, to analyse factors influencing spatial distribution of water resources at the CONUS scale. For example, by combining it with datasets like Gridded Surface Meteorological (GRIDMET), researchers may advance a deeper understanding of how climate conditions influence water resources.
3. Given that streamflow statistics and changepoints at hydrologic stations span the entire river channel within diverse watersheds, our datasets can be instrumental in analyse of environmental factors and hydraulic properties (e.g., river length, width, and skewness) that influence streamflow variations from upstream to downstream. This can be accomplished by integrating our dataset with other relevant sources, such as the National Hydrography Dataset.
4. The two datasets derived from our study are valuable resource for monitoring long-term changes in water resources over the CONUS. Utilizing information on parameters such as the rate of change, variation of streamflow, changepoint, and outliers, researchers can gain insights into local water resource dynamics. Furthermore, the dataset highlights the duration and frequency of low and high flow events at different levels, providing valuable information on extreme events. These indices can assist local water managers in devising more reliable measures to mitigate flood risks (e.g., floodplain and stream restoration).
5. Complementing global river network datasets such as RiverAtlas^40^, our dataset places emphasis on ecological statistics and fundamental properties of hydrological conditions. Leveraging the comprehensive hydro-environmental attributes inherent in river network datasets, our dataset offers new insights into the trends and regime shifts at specific locations.

### Supplementary information

SI for

## Data Availability

All the codes for processing the streamflow metrics and changepoints were calculated using R version 4.3.1 and archived at GitHub: https://github.com/ymwang4924/nsd.
