# Supplementary material for: Streamflow in the United States: Characteristics, trends, regime shifts, and extremes
Source: Sci Data. 2024 Jul 17;11:788. doi: 10.1038/s41597-024-03618-0 (PMC11255205; doi:10.1038/s41597-024-03618-0)
Supplement: Supplementary file 1 — SI for [file 41597_2024_3618_MOESM1_ESM.docx]

**Supplementary Information**

Table of Contents

Fig. S1. Comparison between high flow durations derived from this study using EFLOW and the US flood database (USFD)

Table S1. Definition of streamflow metrics

Table S2. Parameter settings of the BEAST modelling

References


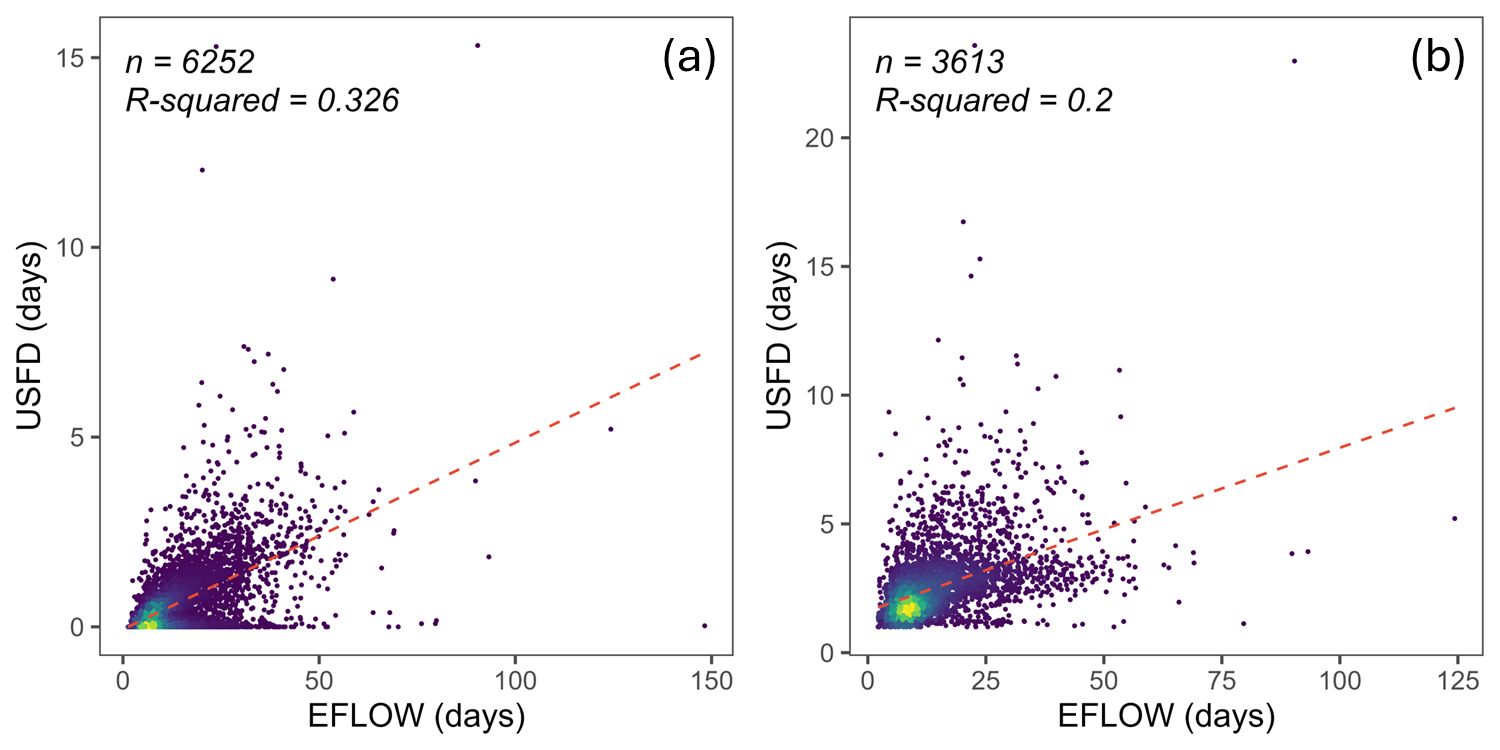


Fig. S1. Comparison between high flow durations derived from this study using EFLOW and the US flood database (USFD) ^1^. All paired records are used in sub-figure (a), while the records with only flood durations greater than 24 hours in USFD are in sub-figure (b). The threshold to identify high flow durations is the 75^th^ percentile (dh21 in Streamflow characteristics dataset).

In comparison with the USFD, considering the spatial proximity of ground-based flooding points to USGS gauges, we excluded flood events located more than 1 km away from a river. Subsequently, each flooding point was matched with the nearest USGS gauge. This process resulted in 6252 matched pairs, with an R2 value of 0.326 for flood durations (Fig. S1a). Then, as USGS daily flow data is used in our study, we compared flood durations exceeding 24 hours and computed the long-term average duration for each matched hydrological station (Fig. S1b). Overall, despite the bias arising from different temporal resolutions and data sources, a modest positive correlation exists between high flow durations from the ground-based flooding data and our daily streamflow dataset.

Table S1. Definition of streamflow metrics

| Code | Definition | Unit |
| --- | --- | --- |
| MA1 | Mean of the daily mean flow values for the entire flow record | cubic feet per second |
| MA2 | Median of the daily mean flow values for the entire flow record | cubic feet per second |
| MA3 | Mean of the coefficients of variation (standard deviation/mean) for each year | percent |
| MA4 | Standard deviation of the percentiles of the logs of the entire flow record divided by the mean of percentiles of the logs | percent |
| MA5 | The skewness of the entire flow record | dimensionless |
| MA6 | Range in daily flows is the ratio of the 10 percent to 90 percent exceedance values for the entire flow record | dimensionless |
| MA7 | Range in daily flows is computed like MA6 except using the 20 percent and 80 percent exceedance values | dimensionless |
| MA8 | Range in daily flows is computed like MA6 except using the 25 percent and 75 percent exceedance values | dimensionless |
| MA9 | Spread in daily flows is the ratio of the difference between the 90th and 10th percentile of the logs of the flow data to the log of the median of the entire flow record | dimensionless |
| MA10 | Spread in daily flows is computed like MA9 except using the 20th and 80th percentiles | dimensionless |
| MA11 | Spread in daily flows is computed like MA9 except using the 25th and 75th percentiles | dimensionless |
| MA12 | Means of monthly flow values in January | cubic feet per second |
| MA13 | Means of monthly flow values in February | cubic feet per second |
| MA14 | Means of monthly flow values in March | cubic feet per second |
| MA15 | Means of monthly flow values in April | cubic feet per second |
| MA16 | Means of monthly flow values in May | cubic feet per second |
| MA17 | Means of monthly flow values in June | cubic feet per second |
| MA18 | Means of monthly flow values in July | cubic feet per second |
| MA19 | Means of monthly flow values in August | cubic feet per second |
| MA20 | Means of monthly flow values in September | cubic feet per second |
| MA21 | Means of monthly flow values in October | cubic feet per second |
| MA22 | Means of monthly flow values in November | cubic feet per second |
| MA23 | Means of monthly flow values in December | cubic feet per second |
| MA24 | Variability (coefficient of variation) of monthly flow values in January | percent |
| MA25 | Variability (coefficient of variation) of monthly flow values in February | percent |
| MA26 | Variability (coefficient of variation) of monthly flow values in March | percent |
| MA27 | Variability (coefficient of variation) of monthly flow values in April | percent |
| MA28 | Variability (coefficient of variation) of monthly flow values in May | percent |
| MA29 | Variability (coefficient of variation) of monthly flow values in June | percent |
| MA30 | Variability (coefficient of variation) of monthly flow values in July | percent |
| MA31 | Variability (coefficient of variation) of monthly flow values in August | percent |
| MA32 | Variability (coefficient of variation) of monthly flow values in September | percent |
| MA33 | Variability (coefficient of variation) of monthly flow values in October | percent |
| MA34 | Variability (coefficient of variation) of monthly flow values in November | percent |
| MA35 | Variability (coefficient of variation) of monthly flow values in December | percent |
| MA36 | Variability across monthly flows. MA36 is the maximum monthly flow minus the minimum monthly flow divided by the median monthly flow | dimensionless |
| MA37 | Variability across monthly flows. MA37 is the third quartile minus the first quartile divided by the median of the monthly means | dimensionless |
| MA38 | Variability across monthly flows. MA38 is the 90th percentile minus the 10th percentile divided by the median of the monthly means | dimensionless |
| MA39 | Variability across monthly flows. MA39 is the standard deviation times 100 divided by the mean of the monthly means | percent |
| MA40 | Skewness in the monthly flows. MA40 is the mean of the monthly flow means minus the median of the monthly means divided by the median of the monthly means | dimensionless |
| MA41 | Annual runoff. | cubic feet per second/square mile |
| MA42 | Variability across annual flows. MA42 is the maximum annual flow minus the minimum annual flow divided by the median annual flow | dimensionless |
| MA43 | Variability across annual flows. MA43 is the third quartile minus the first quartile divided by the median of the annual means | dimensionless |
| MA44 | Variability across annual flows. MA44 is the 90th percentile minus the 10th percentile divided by the median of the annual means | dimensionless |
| MA45 | Skewness in the annual flows. | dimensionless |
| ML1 | Mean of the minimums of all January flow values over the entire record | cubic feet per second |
| ML2 | Mean of the minimums of all February flow values over the entire record | cubic feet per second |
| ML3 | Mean of the minimums of all March flow values over the entire record | cubic feet per second |
| ML4 | Mean of the minimums of all April flow values over the entire record | cubic feet per second |
| ML5 | Mean of the minimums of all May flow values over the entire record | cubic feet per second |
| ML6 | Mean of the minimums of all June flow values over the entire record | cubic feet per second |
| ML7 | Mean of the minimums of all July flow values over the entire record | cubic feet per second |
| ML8 | Mean of the minimums of all August flow values over the entire record | cubic feet per second |
| ML9 | Mean of the minimums of all September flow values over the entire record | cubic feet per second |
| ML10 | Mean of the minimums of all October flow values over the entire record | cubic feet per second |
| ML11 | Mean of the minimums of all November flow values over the entire record | cubic feet per second |
| ML12 | Mean of the minimums of all December flow values over the entire record | cubic feet per second |
| ML13 | Variability (coefficient of variation) across minimum monthly flow values. | percent |
| ML14 | Mean of the ratios of minimum annual flows to the median flow for each year | dimensionless |
| ML15 | Low flow index. ML15 is the mean of the ratios of minimum annual flows to the mean flow for each year | dimensionless |
| ML16 | Median annual minimum flows. ML16 is the median of the ratios of minimum annual flows to the median flow for each year | dimensionless |
| ML17 | Base flow. Compute the minimum of a 7-day moving average flow for each year and divide them by the mean annual flow for that year. ML17 is the mean of those ratios | dimensionless |
| ML18 | Variability in base flow. Compute the standard deviation for the ratios of 7-day moving average flows to mean annual flows for each year. ML18 is the standard deviation times 100 divided by the mean of the ratios | dimensionless |
| ML19 | Base flow. Compute the ratios of the minimum annual flow to mean annual flow for each year. ML19 is the mean of these ratios’ times 100 | dimensionless |
| ML20 | Base flow. ML20 is the ratio of total flow to total base flow | dimensionless |
| ML21 | Variability across annual minimum flows. ML21 is the standard deviation times 100 divided by the mean | percent |
| ML22 | Specific mean annual minimum flow. ML22 is the mean of the annual minimum flows divided by the drainage area | cubic feet per second/square mile |
| MH1 | Mean of the maximums of all January flow values over the entire record | cubic feet per second |
| MH2 | Mean of the maximums of all February flow values over the entire record | cubic feet per second |
| MH3 | Mean of the maximums of all March flow values over the entire record | cubic feet per second |
| MH4 | Mean of the maximums of all April flow values over the entire record | cubic feet per second |
| MH5 | Mean of the maximums of all May flow values over the entire record | cubic feet per second |
| MH6 | Mean of the maximums of all June flow values over the entire record | cubic feet per second |
| MH7 | Mean of the maximums of all July flow values over the entire record | cubic feet per second |
| MH8 | Mean of the maximums of all August flow values over the entire record | cubic feet per second |
| MH9 | Mean of the maximums of all September flow values over the entire record | cubic feet per second |
| MH10 | Mean of the maximums of all October flow values over the entire record | cubic feet per second |
| MH11 | Mean of the maximums of all November flow values over the entire record | cubic feet per second |
| MH12 | Mean of the maximums of all December flow values over the entire record | cubic feet per second |
| MH13 | Variability (coefficient of variation) across maximum monthly flow values. The standard deviation times 100 divided by the mean maximum monthly flow for all years | percent |
| MH14 | Median of annual maximum flows. the median of ratios of annual maximum flow to median annual flow for each year | dimensionless |
| MH15 | High flow discharge index. the 1 percent exceedance value divided by the median flow for the entire record | dimensionless |
| MH16 | High flow discharge index. the 10 percent exceedance value divided by the median flow for the entire record | dimensionless |
| MH17 | High flow discharge index. the 25 percent exceedance value divided by the median flow for the entire record | dimensionless |
| MH18 | Variability across annual maximum flows. the standard deviation times 100 divided by the mean | percent |
| MH19 | Skewness in annual maximum flows | dimensionless |
| MH20 | Specific mean annual maximum flow. the mean of the annual maximum flows divided by the drainage area | cubic feet per second/square mile |
| MH21 | High flow volume index. the average volume divided by the median flow for the entire record | days |
| MH22 | High flow volume. the average volume for flow events above a threshold equal to three times the median flow, then divided by the median flow for the entire record | days |
| MH23 | High flow volume. the average volume for flow events above a threshold equal to seven times the median flow, then divided by the median flow for the entire record | days |
| MH24 | High peak flow. the average peak flow above a threshold equal to the median flow divided by the median flow for the entire record | dimensionless |
| MH25 | High peak flow. the average peak flow above a threshold equal to three times the median flow divided by the median flow for the entire record | dimensionless |
| MH26 | High peak flow. the average peak flow above a threshold equal to seven times the median flow divided by the median flow for the entire record | dimensionless |
| MH27 | High peak flow. the average peak flow above a threshold equal to 75th percentile value divided by the median flow for the entire record | dimensionless |
| FL1 | Low flood pulse count. FL1 is the average number of events with flows below a threshold equal to the 25th percentile value for the entire flow record | number of events/year |
| FL2 | Variability in low pulse count. FL2 is 100 times the standard deviation divided by the mean pulse count | percent |
| FL3 | Frequency of low pulse spells. FL3 is the average number of events with flows below a threshold equal to 5 percent of the mean flow value for the entire flow record. | number of events/year |
| FH1 | High flood pulse count. FH1 is the average number of events with flows above a threshold equal to the 75th percentile value for the entire flow record. | number of events/year |
| FH2 | Variability in high pulse count. FH2 is 100 times the standard deviation divided by the mean pulse count | number of events/year |
| FH3 | High flood pulse count. FH3 is the mean of the annual number of days above a threshold equal to three times the median flow for the entire record for all years. | number of events/year |
| FH4 | High flood pulse count. FH4 is the meanof the annual number of days above a threshold equal to seven times the median flow for the entire record for all years | number of events/year |
| FH5 | Flood frequency. FH5 is the average number of events above a threshold equal to the median flow value for the entire flow record | number of events/year |
| FH6 | Flood frequency. FH6 is the average number of events above a threshold equal to three times the median flow value for the entire flow record | number of events/year |
| FH7 | Flood frequency. FH7 is the average number of events above a threshold equal to seven times the median flow value for the entire flow record. | number of events/year |
| FH8 | Flood frequency. FH8 is the average number of events above a threshold equal to 25 percent exceedance value for the entire flow record. | number of events/year |
| FH9 | Flood frequency. FH9 is the average number of events above a threshold equal to 75 percent exceedance value for the entire flow record. | number of events/year |
| FH10 | Flood frequency. FH10 is the average number of events above a threshold equal to median of the annual minima for the entire flow record. | number of events/year |
| FH11 | Flood frequency. FH11 is the average number of events above a threshold equal to flow corresponding to a 1.67-year recurrence interval. | number of events/year |
| DL1 | Annual minimum daily flow. DL1 is the mean of minimum 1-day average flow for each year. | cubic feet per second |
| DL2 | Annual minimum of 3-day moving average flow. | cubic feet per second |
| DL3 | Annual minimum of 7-day moving average flow. | cubic feet per second |
| DL4 | Annual minimum of 30-day moving average flow. | cubic feet per second |
| DL5 | Annual minimum of 90-day moving average flow. | cubic feet per second |
| DL6 | Variability of annual minimum daily average flow. DL6 is 100 times the standard deviation divided by the mean | percent |
| DL7 | Variability of annual minimum of 3-day moving average flow. | percent |
| DL8 | Variability of annual minimum of 7-day moving average flow. | percent |
| DL9 | Variability of annual minimum of 30-day moving average flow. | percent |
| DL10 | Variability of annual minimum of 90-day moving average flow. | percent |
| DL11 | Annual minimum daily flow divided by the median for the entire record. DL11 is the mean of the minimum daily flow for each year divided by the median for the entire record | dimensionless |
| DL12 | Annual minimum of 7-day moving average flow divided by the median for the entire record. | dimensionless |
| DL13 | Annual minimum of 30-day moving average flow divided by the median for the entire record. | dimensionless |
| DL14 | Low exceedence flows. DL14 is the 75 percent exceedence value for the entire flow record divided by the median for the entire record | dimensionless |
| DL15 | Low exceedence flows. DL15 is the 90 percent exceedence value for the entire flow record divided by the median for the entire record | dimensionless |
| DL16 | Low flow pulse duration. DL16 is the median of the yearly average durations | number of days |
| DL17 | Variability in low pulse duration. DL17 is 100 times the standard deviation divided by the mean of the yearly average low pulse durations | percent |
| DL18 | Number of zero-flow days. | number of days/year |
| DL19 | Variability in the number of zero-flow days. | percent |
| DL20 | Number of zero-flow months. | percent |
| DH1 | Annual maximum daily flow. | cubic feet per second |
| DH2 | Annual maximum of 3-day moving average flows. | cubic feet per second |
| DH3 | Annual maximum of 7-day moving average flows. | cubic feet per second |
| DH4 | Annual maximum of 30-day moving average flows. | cubic feet per second |
| DH5 | Annual maximum of 90-day moving average flows. | cubic feet per second |
| DH6 | Variability of annual maximum daily flows. DH6 is 100 times the standard deviation divided by the mean | percent |
| DH7 | Variability of annual maximum of 3-day moving average flows. | percent |
| DH8 | Variability of annual maximum of 7-day moving average flows. | percent |
| DH9 | Variability of annual maximum of 30-day moving average flows. | percent |
| DH10 | Variability of annual maximum of 90-day moving average flows. | percent |
| DH11 | Annual maximum of 1-day moving average flows divided by the median for the entire record. | dimensionless |
| DH12 | Annual maximum of 7-day moving average flows divided by the median for the entire record. | dimensionless |
| DH13 | Annual maximum of 30-day moving average flows divided by the median for the entire record. | dimensionless |
| DH14 | Flood duration. DH14 is the 95th percentile value divided by the mean of the monthly means | dimensionless |
| DH15 | High flow pulse duration. DH15 is the median of the yearly average durations for flow events with flows above a threshold equal to the 75th percentile value for each year in the flow record | days/year |
| DH16 | Variability in high flow pulse duration. DH16 is 100 times the standard deviation divided by the mean of the yearly average high pulse durations | percent |
| DH17 | High flow duration. DH17 is the average duration of the events with flows above a threshold equal to the median flow value for the entire flow record | days |
| DH18 | High flow duration. DH18 is the average duration of the events with flows above a threshold equal to three times the median flow value for the entire flow record | days |
| DH19 | High flow duration. DH19 is the average duration of the events with flows above a threshold equal to seven times the median flow value for the entire flow record | days |
| DH20 | High flow duration. DH20 is the average duration of the events with flows above a threshold equal to the 75th percentile value for the median annual flows | days |
| DH21 | High flow duration. DH21 is the average duration of the events with flows above a threshold equal to the 25th percentile value for the entire set of flows | days |
| DH22 | Flood interval. Compute the flood threshold as the flow equivalent for a flood recurrence of 1.67 years. DH22 is the mean of the yearly median number of days between flood events | days |
| DH23 | Flood duration. Compute the flood threshold as the flow equivalent for a flood recurrence of 1.67 years. DH23 is the mean of the number of flood days for years in which floods occur | days |
| DH24 | Flood-free days. DH24 is the mean of the maximum yearly no-flood days | days |
| TA1 | Constancy. Constancy is computed via the formulation of Colwell (see example in Colwell, 1974). | dimensionless |
| TA2 | Predictability. Predictability is computed from the same matrix as constancy (see example in Colwell, 1974). | dimensionless |
| TA3 | Seasonal predictability of flooding. | dimensionless |
| TL1 | Julian date of annual minimum. | Julian day |
| TL2 | Variability in Julian date of annual minima. | Julian day |
| TL3 | Seasonal predictability of low flow. | dimensionless |
| TL4 | Seasonal predictability of non-low flow. | dimensionless |
| TH1 | Julian date of annual maximum. | Julian day |
| TH2 | Variability in Julian date of annual maxima. | Julian day |
| TH3 | Seasonal predictability of nonflooding. | dimensionless |
| RA1 | Rise rate. RA1 is the mean of the change in flow for days in which the change is positive for the entire flow record | cubic feet per second/day |
| RA2 | Variability in rise rate. RA2 is 100 times the standard deviation divided by the mean | percent |
| RA3 | Fall rate. RA3 is the mean of the change in flow for days in which the change is negative for the entire flow record. | cubic feet per second/day |
| RA4 | Variability in fall rate. RA4 is 100 times the standard deviation divided by the mean | percent |
| RA5 | Number of day rises. RA5 is the number of positive gain days divided by the total number of days in the flow record | dimensionless |
| RA6 | Change of flow. RA6 is the median of the change in log10 of flow for days in which the change is positive for the entire flow record | cubic feet per second |
| RA7 | Change of flow. RA7 is the median of the change in log10 of flow for days in which the change is negtive for the entire flow record | cubic feet per second/day |
| RA8 | Number of reversals. RA8 is the average of the number of days in each year when the change in flow from one day to the next changes direction | days |
| RA9 | Variability in reversals. RA9 is 100 times the standard deviation divided by the mean | percent |
| lam1 | mean | cubic feet per second |
| tau2 | coefficient of variation | dimensionless |
| tau3 | skewness | dimensionless |
| tau4 | kurtosis | dimensionless |
| ar1 | autoregressive lag-one correlation coefficient | dimensionless |
| amplitude | amplitude | cubic feet per second |
| phase | phase of the seasonal signal | dimensionless |
| X5. | 5% quantile of streamflow for the entire flow record | cubic feet per second |
| X95. | 95% quantile of streamflow for the entire flow record | cubic feet per second |

Table S2. Parameter settings of the BEAST modelling

| **Group** | **Parameter** | **Description** | **Value** |
| --- | --- | --- | --- |
| **metadata** | season | Fit a harmonic model to the periodic component | svd |
|  | deltaTime | The time interval between consecutive data points | 1/12 year |
|  | period | The period for the periodic/seasonal component | 1 |
|  | maxMissingRate | If more than maxMissingRate of data is missing, BEAST will skip it | 0.75 |
|  | hasOutlier |  | TRUE |
| **prior** | seasonMinOrder | Min harmonic order alllowed | 1 |
|  | seasonMaxOrder | Max harmonic order alllowed | 5 |
|  | seasonMinKnotNum | Min num of seasonal changepoints | 0 |
|  | seasonMaxKnotNum | Max num of seasonal changepoints | 10 |
|  | seasonMinSepDist | Min seasonal segment length in terms of datapoints | 24 |
|  | trendMinOrder | Min trend polynomial order alllowed | 0 |
|  | trendMaxOrder | Max trend polynomial order alllowed | 1 |
|  | trendMinKnotNum | Min number of changepoints in trend | 0 |
|  | trendMaxKnotNum | Min number of changepoints in trend | 10 |
|  | trendMinSepDist | Min trend segment length in terms of datapoints | 24 |
|  | precValue | The hyperparameter of the precision prior | 1.5 |
|  | precPriorType | The precision parameter used to parameterize the model coefficients is a random variable | uniform |
|  | outlierMaxKnotNum | Max number of seasonal changepoints | 20 |
| **mcmc** | seed | A nonzero seed to replicate among runs | 0 |
|  | samples | Number of samples saved per chain | 8000 |
|  | thinningFactor | Thinning the chain | 5 |
|  | burnin | Number of initial samples discarded | 200 |
|  | chainNumber | Number of chains | 3 |
|  | maxMoveStepSize | Max step of jumping from current changepoint | 12 |
|  | trendResamplingOrderProb | Proposal probability of sampling trend polynominal order | 0.1 |
|  | seasonResamplingOrderProb | Proposal probability of sampling seasonal order | 0.17 |
|  | credIntervalAlphaLevel | The alpha level for Credible Intervals | 0.95 |

References

1. Li, Z. *et al.* A multi-source 120-year US flood database with a unified common format and public access. *Earth System Science Data Discussions* **2021**, 1–25 (2021).
